# Performance Analysis in the Decode-and-Forward Full-Duplex Relaying Network with SWIPT

**DOI:** 10.1155/2021/5548802

**Published:** 2021-04-16

**Authors:** Phu Tran Tin, Phan Van-Duc, Tan N. Nguyen, Le Anh Vu

**Affiliations:** ^1^Faculty of Electronics Technology, Industrial University of Ho Chi Minh City, Ho Chi Minh City, Vietnam; ^2^Faculty of Automobile Technology, Van Lang University, Ho Chi Minh City, Vietnam; ^3^Wireless Communications Research Group, Faculty of Electrical and Electronics Engineering, Ton Duc Thang University, Ho Chi Minh City, Vietnam; ^4^Optoelectronics Research Group, Faculty of Electrical and Electronics Engineering, Ton Duc Thang University, Ho Chi Minh City, Vietnam

## Abstract

This paper investigates the decode-and-forward (DF) full-duplex (FD) cooperative relaying system with SWIPT. Specifically, the relay node can harvest energy from the source's RF signal, and then the harvested energy is used for transferring information to the destination. Besides, we consider both direct and two-hop relaying links to transmit data from the source to the destination. In the performance analysis, we derive the exact expressions for outage probability (OP) by applying the receiver's selection combining (SC) technique. Then, the Monte Carlo simulation is performed to verify the correctness of the mathematical analysis. Finally, the simulations show that the mathematic expressions match simulation results, which authenticates the mathematical analysis.

## 1. Introduction

Recently, the explosive growth of the Internet of Things (IoT) has led to a massive amount of traffic data, which has brought a great burden on the mobile devices' energy consumption [1–5]. Presently, most device users are equipped with a limited onboard battery; thus, their batteries need to be recharged or replaced periodically, which is difficult and infeasible in some cases, e.g., in the human body or in harsh environments. Consequently, scavenging energy from surrounding environments such as solar, wind, water, and heat enhances the batteries' endurance [6–19]. However, these methods heavily depend on uncontrollable elements such as geographic location and weather conditions, which do not guarantee a stable energy source. Fortunately, wireless power transfer (WPT) can be considered as a promising solution. Particularly, since the RF signals can contain both energy and information, simultaneous transmit information and energy transfer (SWIPT) has recently attracted significant attention from researchers [20, 21]. Qian et al. [20] studied a buffer-aided differential chaos-shift-keying-based simultaneous wireless information and power transfer (DCSK-SWIPT) relay system over multipath Rayleigh fading channels. By using Meijer G-function and Gauss–Hermite approach, they obtained bit error rate (BER) and average-delay closed-form expressions. Akash et al. [21] considered a NOMA SWIPT-enabled cooperative communication system with a finite blocklength (FBL) twin-user. Based on the proposed model, novel closed-form analytical expressions for the end-to-end average block error rate (BLER) are obtained.

Besides energy harvesting, full-duplex technology for cooperative relaying systems also attracted significant attention from researchers [22–24]. In [22], the authors considered a novel system model consisting of an energy-harvesting full-duplex (FD) relay and a jammer in the presence of an eavesdropper. To improve the energy efficiency and security, they proposed two methods, namely, full-duplex and half-duplex jammer protocols. Tan et al. [23] proposed a new decode-and-forward (DF) FD relaying network over the Rician channels. Then, they derived closed-form expressions of the throughput, OP, and symbol error rate (SER). In contrast to [22, 23] that only investigated the single-input single-output (SISO) system, Zhao et al. [24] studied physical layer security for a full-duplex multiple-input multiple-output system.

Motivated by the above discussions, this paper proposed and investigated the system performance analysis of a SWIPT-aided relay network in the full-duplex (FD) decode-and-forward (DF) mode with the consistency of direct link from the selection source to a destination. Moreover, the relay node is equipped with a full-duplex antenna, and it can harvest energy from the source node. The contributions of this paper are listed as follows:

  We model a novel SWIPT-enabled DF relaying network with full-duplex transmission in the presence of direct link to improve the total throughput at the destination.
  Based on the proposed system model, we derive the exact closed-form expression of outage probability at the destination.
  The correctness of the mathematical analysis is validated through Monte Carlo simulations. Specifically, the influences of different system parameters on the system performance are investigated, i.e., number of sources, rate threshold requirement, source transmit power, and power splitting ratio.

The remainder of this paper is organized as follows. In Section 2, the system model of the decode-and-forward full-duplex relay network with SWIPT is described in detail. Then, in Section 3, we provide the outage probability analysis of the system. Numerical results to support our analysis are presented in Section 4. Finally, Section 5 concludes the paper.

## 2. System Model

**Figure 1:**
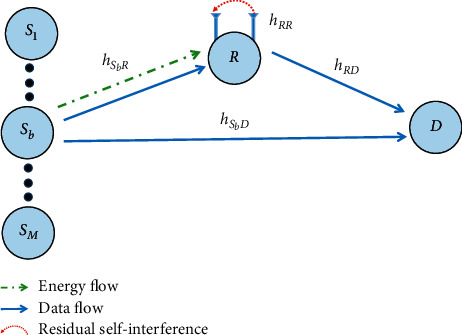
System model.

**Figure 2:**
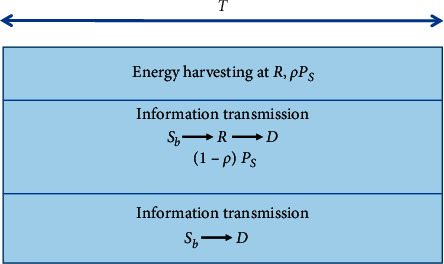
IT and EH processes.

We consider a cooperative relaying network as shown in Figure 1, where a relay *R* helps transfer data from a source *S*_*b*_ to a destination *D*. In particular, a destination *D* can get information from the source and relay by applying the selection combining (SC) technique. Besides, source *S*_*b*_ and destination are equipped with a single antenna and operate in the HD mode, while the relay *R* has two antennas and operates in the FD mode. In Figure 2, the relay *R* can harvest energy by adopting the power splitting method. Specifically, a fraction of power *ρP*_*S*_ is used for energy harvesting, and the rest (1 − *ρ*)*P*_*S*_ is used for information decoding.

The channel between two users is assumed to be block Rayleigh fading, where channel gain is a constant value during one block and changes across different blocks. Moreover, the channel coefficient between node *X* and node *Y* can be expressed as *h*_*XY*_ for *XY*  ∈ {*SR*, *RD*, *RR*, *SD*, *RE*, *SE*}. Besides, the squared amplitudes of the channel gains such as *|h*_*SD*_*|*^2^ and *|h*_*RD*_*|*^2^ are exponential random variables (RVs).

The received signal at the relay is given as follows:

(1) $$\begin{array}{c} y_{R}=\sqrt{1-\rho }h_{S_{b}R}x_{s}+h_{RR}x_{R}+n_{R}, \end{array}$$

where *x*_*S*_ is the source's transmit symbol and *E*{|*x*_*s*_|^2^}=*P*_*s*_,  *x*_*R*_ is the self-interference due to full-duplex (FD) relaying and satisfies *E*{|*x*_*R*_|^2^}=*P*_*R*_, where Ε{•}is the expectation operation. Besides, *h*_*RR*_ and *n*_*R*_ denote the loop-back interference channel and zero-mean additive white Gaussian noise (AWGN) with variance *N*_0_, respectively.

The relay's harvested energy is calculated as

(2) $$\begin{array}{c} E_{R}=\eta \rho TP_{s}{\left|h_{S_{b}R}\right|}^{2}. \end{array}$$

From (2), the average transmit power of the relay node is given by

(3) $$\begin{array}{c} P_{R}=\frac{E_{R}}{T}=\eta \rho P_{s}{\left|h_{S_{b}R}\right|}^{2}, \end{array}$$

where 0 < *η* ≤ 1 denotes the energy conversion efficiency, which takes into account the energy loss by harvesting circuits and also by decoding and processing circuits.

The received signal at the destination from the source and relay is, respectively, given by

(4) $$\begin{array}{c} y_{D}^{1}=h_{S_{b}D}x_{s}+n_{D}^{1},y_{D}^{2}=h_{RD}x_{R}+n_{D}^{2}, \end{array}$$

where *n*_*D*_^1^=*n*_*D*_^2^= *n*_*D*_ denotes the AWGN with variance *N*_0_ at the destination *D*.

In our model, we adopt the decode-and-forward (DF) protocol. Consequently, the signal-to-interference-to-noise ratio (SINR) at the relay *R* can be given by

(5) $$\begin{array}{c} \gamma_{R}=\frac{\left(1-\rho \right)P_{s}{\left|h_{S_{b}R}\right|}^{2}}{{\left|h_{RR}\right|}^{2}P_{R}+N_{0}}. \end{array}$$

Substituting (3) into (5) and using the fact that *N*_0_ << *P*_*S*_, we have

(6) $$\begin{array}{c} \gamma_{R}=\frac{\left(1-\rho \right)P_{s}{\left|h_{SR}\right|}^{2}}{\eta \rho P_{s}{\left|h_{SR}\right|}^{2}{\left|h_{RR}\right|}^{2}+N_{0}}\approx \frac{1-\rho }{\eta \rho {\left|h_{RR}\right|}^{2}}. \end{array}$$

From (4), the SINR at the destination is expressed as

(7) $$\begin{array}{c} \gamma_{D}^{1}=\frac{{\left|h_{S_{b}D}\right|}^{2}P_{s}}{N_{0}}=\Phi {\left|h_{S_{b}D}\right|}^{2},\\ \gamma_{D}^{2}=\frac{{\left|h_{RD}\right|}^{2}P_{R}}{N_{0}}=\frac{\eta \rho P_{s}{\left|h_{S_{b}R}\right|}^{2}{\left|h_{RD}\right|}^{2}}{N_{0}}=\eta \rho \Phi {\left|h_{S_{b}R}\right|}^{2}{\left|h_{RD}\right|}^{2}, \end{array}$$

where Φ=*P*_*s*_/*N*_0_.

For simplicity, we assume that *D* uses the selection combining (SC) technique. Consequently, the overall SINR of the system can be expressed as

(8) $$\begin{array}{c} \gamma_{e2e}=\max\left(\gamma_{DF},\gamma_{D}^{1}\right), \end{array}$$

where

(9) $$\begin{array}{c} \gamma_{DF}=\min\left(\gamma_{R},\gamma_{D}^{2}\right)=\min\left(\frac{1-\rho }{\eta \rho {\left|h_{RR}\right|}^{2}},\eta \rho \Phi {\left|h_{S_{b}R}\right|}^{2}{\left|h_{RD}\right|}^{2}\right). \end{array}$$

**Remark 1 .**

We propose the optimal source selection protocol, in which the best selection source is given as follows:

(10) $$\begin{array}{c} b=\arg\underset{1\leq b\leq M}{\max}\left[{\left|h_{S_{b}D}\right|}^{2}\right]. \end{array}$$

By denoting $X=\underset{b=1,2,\ldots ,M}{\max}\left({\left|h_{S_{b}D}\right|}^{2}\right)$, the cumulative distribution function (CDF) of *X* can be given by

(11) $$\begin{array}{c} F_{X}\left(x\right)=\overset{M}{\underset{b=0}{\sum }}{\left(-1\right)}^{b}C_{M}^{b}\times \exp\left(-\lambda_{S_{b}D}bx\right)=1+\overset{M}{\underset{b=1}{\sum }}{\left(-1\right)}^{b}C_{M}^{b}\times \exp\left(-\lambda_{S_{b}D}bx\right), \end{array}$$

where *C*_*M*_^*b*^=*M*!/*b*!(*M* − *b*)! and *λ*_*S*_*b*_*D*_ is the mean of random variable (RV)|*h*_*S*_*b*_*D*_|^2^.

Then, the corresponding probability density function (PDF) can be obtained by

(12) $$\begin{array}{c} f_{X}\left(x\right)=\lambda_{S_{b}D}\overset{M-1}{\underset{b=0}{\sum }}{\left(-1\right)}^{b}C_{M-1}^{b}M\times \exp\left[-\lambda_{S_{b}D}\left(b+1\right)x\right]. \end{array}$$

## 3. Outage Probability (OP) Analysis

The OP of the system can be defined as [6]

(13) $$\begin{array}{c} OP=\mathrm{Pr}\left(\gamma_{e2e}<\gamma_{th}\right), \end{array}$$

where *γ*_*th*_=2^*R*^ − 1 is the system threshold to decode the signal successfully and *R* is the data transmission rate.

By combining with (6)–(8), the OP can be calculated as

(14) $$\begin{array}{c} OP=\mathrm{Pr}\left(\max\left(\gamma_{DF},\gamma_{D}^{1}\right)<\gamma_{th}\right)\\ =\mathrm{Pr}\left\{\max\left(\min\left(1-\rho /\eta \rho {\left|h_{RR}\right|}^{2},\eta \rho \Phi {\left|h_{S_{b}R}\right|}^{2}{\left|h_{RD}\right|}^{2}\right),\Phi \max\left({\left|h_{S_{b}D}\right|}^{2}\right)\right)<\gamma_{th}\right\}\\ =\underset{P_{1}}{\underset{︸}{\mathrm{Pr}\left(\min\left(1-\rho /\eta \rho {\left|h_{RR}\right|}^{2},\eta \rho \Phi {\left|h_{S_{b}R}\right|}^{2}{\left|h_{RD}\right|}^{2}\right)<\gamma_{th}\right)}}\times \underset{P_{2}}{\underset{︸}{\mathrm{Pr}\left(\Phi \max\left({\left|h_{S_{b}D}\right|}^{2}\right)<\gamma_{th}\right)}}. \end{array}$$

Specifically, *P*_1_ in (14) can be computed as

(15) $$\begin{array}{c} P_{1}=1-\underset{P_{11}}{\underset{︸}{\mathrm{Pr}\left(1-\rho /\eta \rho {\left|h_{RR}\right|}^{2}\geq \gamma_{th}\right)}}\times \underset{P_{12}}{\underset{︸}{\mathrm{Pr}\left(\eta \rho \Phi {\left|h_{S_{b}R}\right|}^{2}{\left|h_{RD}\right|}^{2}\geq \gamma_{th}\right)}}. \end{array}$$

From (15), *P*_11_ is calculated by

(16) $$\begin{array}{c} P_{11}=\mathrm{Pr}\left(\frac{1-\rho }{\eta \rho Z}\geq \gamma_{th}\right)=\mathrm{Pr}\left(Z\leq \frac{1-\rho }{\eta \rho \gamma_{th}}\right)=1-\exp\left(-\frac{\lambda_{RR}\left(1-\rho \right)}{\eta \rho \gamma_{th}}\right), \end{array}$$

where *Z*=|*h*_*RR*_|^2^ and *λ*_*RR*_ is the mean of RV *Z*.

Next, *P*_12_ is formulated as

(17) $$\begin{array}{c} P_{12}=\mathrm{Pr}\left(\eta \rho \Phi YT\geq \gamma_{th}\right)=1-\mathrm{Pr}\left(\eta \rho \Phi YT<\gamma_{th}\right)\\ =1-\mathrm{Pr}\left(Y<\frac{\gamma_{th}}{\eta \rho \Phi T}\right)=1-\overset{\infty }{\underset{0}{\int }}F_{Y}\left(\frac{\gamma_{th}}{\eta \rho \Phi t}|T=t\right)f_{T}\left(t\right)\text{d}t\\ =\overset{\infty }{\underset{0}{\int }}\lambda_{RD}\exp\left(-\frac{\lambda_{S_{b}R}\gamma_{th}}{\eta \rho \Phi t}-\lambda_{RD}t\right)\text{d}t, \end{array}$$

where *Y*=|*h*_*S*_*b*_*R*_|^2^, *T*=|*h*_*RD*_|^2^, and *λ*_*S*_*b*_*R*_, *λ*_*RD*_ are the mean of RVs *Y* and *T*, respectively.

By applying Eq. 3.324,1 of [25], *P*_12_ can be rewritten as

(18) $$\begin{array}{c} P_{12}=2\sqrt{\frac{\lambda_{S_{b}R}\lambda_{RD}\gamma_{th}}{\eta \rho \Phi }}\times K_{1}\left(2\sqrt{\frac{\lambda_{S_{b}R}\lambda_{RD}\gamma_{th}}{\eta \rho \Phi }}\right), \end{array}$$

where *K*_*v*_(•) is the modified Bessel function of the second kind and *v*-th order.

Substituting (16) and (17) into (15), we have

(19) $$\begin{array}{c} P_{1}=1-2\left\{1-\exp\left(-\frac{\lambda_{RR}\left(1-\rho \right)}{\eta \rho \gamma_{th}}\right)\right\}\times \sqrt{\frac{\lambda_{S_{b}R}\lambda_{RD}\gamma_{th}}{\eta \rho \Phi }}\times K_{1}\left(2\sqrt{\frac{\lambda_{S_{b}R}\lambda_{RD}\gamma_{th}}{\eta \rho \Phi }}\right). \end{array}$$

By combining (11) and (14), *P*_2_ can be expressed by

(20) $$\begin{array}{c} P_{2}=\mathrm{Pr}\left(\max\left({\left|h_{S_{b}D}\right|}^{2}\right)<\frac{\gamma_{th}}{\Phi }\right)=1+\overset{M}{\underset{b=1}{\sum }}{\left(-1\right)}^{b}C_{M}^{b}\times \exp\left(-\frac{\lambda_{S_{b}D}b\gamma_{th}}{\Phi }\right). \end{array}$$

Finally, by substituting (19) and (20) into (14), the OP is represented as

(21) $$\begin{array}{c} OP=\left\{1-2\left\{1-\exp\left(-\frac{\lambda_{RR}\left(1-\rho \right)}{\eta \rho \gamma_{th}}\right)\right\}\times \sqrt{\frac{\lambda_{S_{b}R}\lambda_{RD}\gamma_{th}}{\eta \rho \Phi }}\times K_{1}\left(2\sqrt{\frac{\lambda_{S_{b}R}\lambda_{RD}\gamma_{th}}{\eta \rho \Phi }}\right)\right\}\\ \times \left\{1+\overset{M}{\underset{b=1}{\sum }}{\left(-1\right)}^{b}C_{M}^{b}\times \exp\left(-\frac{\lambda_{S_{b}D}b\gamma_{th}}{\Phi }\right)\right\}. \end{array}$$

## 4. Throughput Analysis

The system throughput can be defined as [26]

(22) $$\begin{array}{c} \tau =\left(1-OP\right)\times R. \end{array}$$

## 5. Simulation Results

This section provides the simulation results to verify the performance, i.e., outage probability (OP) and intercept probability (IP), of our proposed methods with the selection combining (SC) technique. The results are obtained by running 10^6^ Rayleigh channel realizations using Monte Carlo simulations [28–31].

**Figure 3:**
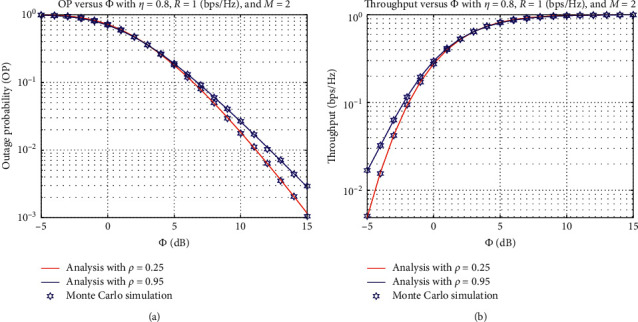
OP and IP versus *ϕ*(dB).

In Figure 3, we investigate the OP and IP as functions of *ϕ*(dB) with different values of *ρ*, where *η*=0.8, rate threshold *R* = 1 bps/Hz, and the number of sources *M* = 2. As observed from Figure 3(a), when we increase the *ϕ*(dB) value, the outage performance at the destination *D* is improved. This is expected since the higher *ϕ* value means that more transmit power is allocated to the selected source*S*_*b*_. It leads to a larger amount of data transmission rate that can be obtained at the destination *D*, which decreases the probability that the outage occurs. Moreover, the proposed power-splitting scheme with *ρ*=0.25 can achieve a better result as compared with that of *ρ*=0.95. From Figure 3(b), we can see that the achievable throughput at the destination *D* is significantly improved with a higher value of *ϕ*(dB). Moreover, the throughput of the proposed scheme with *ρ*=0.95 is better than that as compared with the proposed scheme with *ρ*=0.25 when Φ ≤ 1 dB. However, both schemes can obtain the same throughput when Φ ≥ 2 dB.

**Figure 4:**
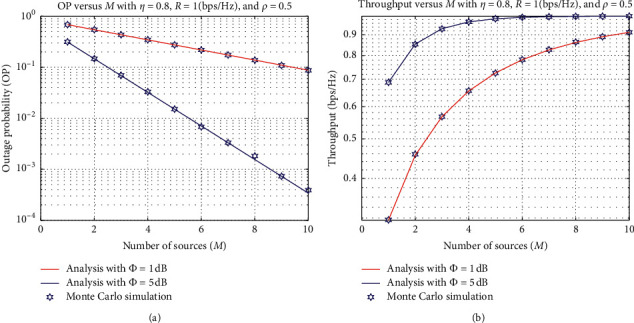
OP and IP versus *M*.

Figure 4 illustrates the OP and IP as functions of the number of sources *M* with different values of *ϕ*(dB), where *η*=0.8, rate threshold *R* = 1 bps/Hz, and *ρ*=0.95. First, we can see that the higher the number of sources is, the better the system performance (i.e., the outage and throughput performances) can be achieved. This can be explained by the fact that we have more choices to select the better source, which maximizes the channel from the source to the destination, as shown in equation (10). Besides, the OP of the proposed scheme with *ϕ*=5 dB is better than that of the proposed scheme with *ϕ*=1 dB, which is shown in Figure 3(a). Another observation from Figure 4(a) is that the OP gap between two schemes is more severe with a higher number of sources. In contrast to Figure 3(a), the IP gap between the two proposed schemes is smaller when the *M* value increases. This is because the total throughput at the destination *D* depends not only on the number of sources but also on the source transmit power and the total transmission time.

**Figure 5:**
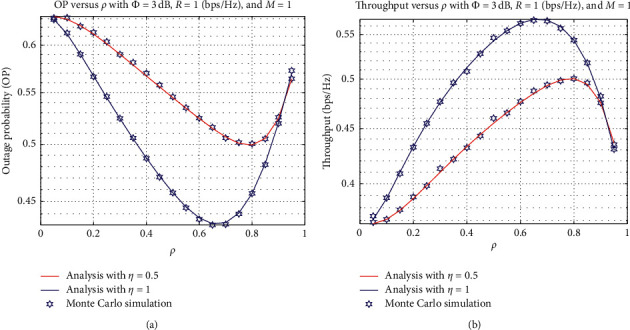
OP and IP versus *ρ*.

Figure 5 shows the OP and IP as functions of *ρ*, with *ϕ*=3 dB, rate threshold *R* = 1 bps/Hz, and the number of sources *M* = 2. *ρ* plays an important role because it influences the allocated time for the harvested energy at the relay, and the energy is used for information decoding. Therefore, the more the value of *α* is, the larger the energy that the relay can harvest. Nevertheless, less time is spent on information transmission from the relay to the destination. It leads to that the OP can obtain the best result at the optimal *ρ* value, and then it is degraded, as shown in Figure 5(a). Based on the OP obtained from Figure 5(a), the throughput performance also increases to an optimal value, and then it decreases. It can be explained based on the definition of throughput as in equation (22). Specifically, the lower the OP value is, the better the throughput can be achieved.

**Figure 6:**
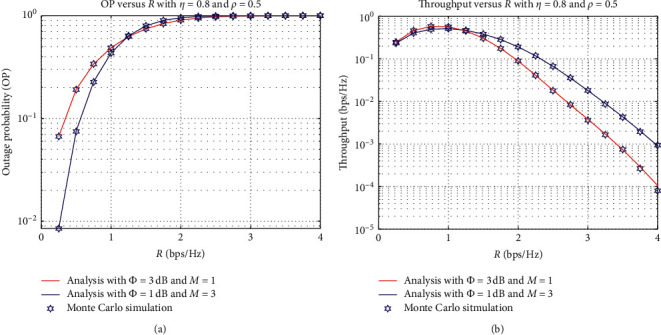
OP and IP versus *R* (bps/Hz).

In Figure 6, we investigate the OP and IP as functions of the rate threshold requirement to decode the signal successfully, where *ρ*=0.5 and *η*=1. Specifically, we consider two schemes: the first one is set as *ϕ*=3 dB and *M* = 1, and the second one is set as *ϕ*=1 dB and *M* = 3. We can see from Figure 6(a) that the OP increases when the rate value increases. It is because the higher the rate requirement is, the data transmission rate received at the destination should be large enough to decode the signal. However, the transmission rate is restricted by received power and allocated time. Furthermore, it can be observed from Figure 6(b) that the total system throughput slightly increases when the rate threshold changes from zero to the optimal value, and then it dramatically decreases. For example, the throughput of the second scheme with *ϕ*=1  dB and *M* = 3 increases from 0.2479 to 0.5793 bps/Hz when *R* ranges from 0.25 to 0.75 bps/Hz. Then, its performance degrades to 0.00008 at *R* = 4 bps/Hz.

## 6. Conclusions and Future Works

This paper studied the DF FD relaying network with a direct link between the source and destination. Specifically, the FD-enabled relay node can harvest energy from the source and transmit data to the destination at the same time. By taking into account the above discussions, we derive the exact closed-form expression of the outage probability (OP) in the receiver's selection combining (SC) technique. Moreover, the simulation results show the correctness of the analytical results compared with the Monte Carlo simulation. For future works, we can extend this study to a more generalized model by considering a nonlinear EH. Another promising problem is to consider two-way relaying networks, which provide higher spectral efficiency.

## Data Availability

No data were used to support this study.
